# Uptake of an Incentive-Based mHealth App: Process Evaluation of the Carrot Rewards App

**DOI:** 10.2196/mhealth.7323

**Published:** 2017-05-30

**Authors:** Marc Mitchell, Lauren White, Paul Oh, David Alter, Tricia Leahey, Matthew Kwan, Guy Faulkner

**Affiliations:** ^1^ University Health Network Toronto, ON Canada; ^2^ Carrot Insights Inc Toronto, ON Canada; ^3^ University of Connecticut Storrs, CT United States; ^4^ McMaster University Hamilton, ON Canada; ^5^ University of British Columbia Vancouver, BC Canada

**Keywords:** financial incentives, mHealth, behavioral economics, public health

## Abstract

**Background:**

Behavioral economics has stimulated renewed interest in financial health incentives worldwide. The Carrot Rewards app was developed as part of a public-private partnership to reward Canadians with loyalty points (eg, movies and groceries) for downloading the app, referring friends, and completing an average of 1 to 2 educational health quizzes per week (“micro-learning”), with long-term objectives of increasing health knowledge and encouraging healthy behaviors.

**Objective:**

The main objective of this study was to evaluate uptake of a loyalty points-based mHealth app during the exclusive 3-month launch period in British Columbia (BC), Canada. The secondary aims were to describe the health and sociodemographic characteristics of users, as well as participation levels (eg, proportion of quizzes completed and friends referred).

**Methods:**

The app was promoted via loyalty program email campaigns (1.64 million emails). Number of downloads and registrations (users enter age, gender, and valid BC postal code to register) were collected. Additional sociodemographics were inferred by linking postal codes with census data at the local health area (LHA) level. Health risk assessments were also deployed. Participation levels were collected over 3 months and descriptive data were presented.

**Results:**

In 3 months, 67,464 individuals downloaded the app; in its first week, Carrot Rewards was the most downloaded health app in Canada. Among valid users (n=57,885; at least one quiz completed), the majority were female (62.96%; 36,446/57,885) and aged 18 to 34 years (54.34%; 31,459/57,885). More than half of the users (52.40%; 30,332/57,885) resided in LHAs where the median personal income was below the provincial average (Can $28,765). Furthermore, 64.42% (37,291/57,885) of users lived in metropolitan (ie, urban) LHAs, compared with 56.17% of the general BC population. The most prevalent risk factors were “not” meeting physical activity guidelines (72.70%; 31,765/43,692) and “not” getting the flu shot last year (67.69%; 30,286/44,739). Regarding participation, 60.05% (34,761/57,885) of users were classified as “very high” engagers (>75% quiz completion rate).

**Conclusions:**

Early results suggest that loyalty points may promote mHealth app uptake. The app was downloaded by younger females especially, and BC residents from higher and lower income regions were equally represented. Loyalty points appear to have driven participation throughout the inaugural 3-month period (ie, quiz completion).

## Introduction

The cost of health care in Canada is rising at an unsustainable rate [1]. Although health care costs are shared between federal, provincial, and territorial governments in Canada, ultimately provinces and territories are responsible for health care spending, organization, delivery, and management of health care services and incur a greater proportion of health care costs. About 40% of provincial and territorial budgets are spent on health care alone, and total health expenditure growth in 2015 was forecasted to be 1.6% [2]—this, at a time when the number of older Canadians at high-risk of developing chronic conditions is on the rise (5 million Canadians aged 65 years+ in 2011 vs 10 million expected in 2036) [3]. Expensive chronic diseases, however, are not just reserved for older adults, as approximately half of Canadians aged 20 years and above live with at least one chronic condition [4]. In the 2014 economic analysis, the costliest modifiable risk factors in Canada were smoking, physical inactivity, and obesity, accounting for Can $50 billion in direct health care expenses [5]. Although improving an individual’s risk factor profile is possible, health behavior change can be extremely difficult. Persisting levels of smoking (18%) [6], physical inactivity (80%) [7], and overweight or obesity (54%) [8] in Canada provides case-in-point. Acknowledging that health behaviors are influenced across multiple domains and require multipronged and multisectoral solutions, governments are looking for new, innovative, and collaborative ways of promoting healthy lifestyles [9,10].

Behavioral economics, a branch of economics complimented by insights from psychology, has stimulated renewed interest in using financial incentives to motivate healthy behaviors on a population scale [11-13]. Briefly, behavioral economics recognizes that human decisions are biased in systematic ways and that these “decision biases” can make it hard for people to make self-beneficial choices [14,15]. For example, people often succumb to the “present bias” when making health-related decisions because they place disproportionate emphasis on the present “costs” of health behaviors (ie, time out of a busy schedule) and discount the future benefits (eg, quality of life). According to behavioral economics, increasing the immediately rewarding aspects of health behaviors (with a financial incentive, for example) may offset the so-called “present bias,” increasing the likelihood of action [14,15]. A growing evidence base seems to support this theoretical rationale. For instance, several systematic reviews and meta-analyses have shown that incentives generally stimulate health behaviors [16-22]. Despite some notable gaps in the literature (eg, long-term effects are not clear), financial health incentives have grown in popularity. The best examples of broad-based application come from Germany and the United States where incentive-based public health policies have been in place since 2004 and 2014, respectively [11,23]. In the United States, the Patient Protection and Affordable Care Act (2010) allows employers (as of the year 2014) to reimburse their employees up to US $1500 per year for engaging in healthy behaviors or reaching health targets [23]. An often-cited limitation of these programs and policies, however, is the notorious delay between (1) behavior or outcome achievement and (2) reward [15,23,24]. Generally speaking, this lag has been too long (and the incentives not large or meaningful enough) to elicit the desired behavioral responses or health outcomes [23,24].

The pervasive use of smartphones in Canada (in 2015, 73% of Canadian adults owned a smartphone—a relative increase of 7% compared with 2014) [25], presents governments with an opportunity to offer financial health incentives on a population scale, with little delay between behavior and reward—leveraging people’s predictable tendency to overvalue the benefits they experience in the present. Moreover, smartphone capabilities have evolved (eg, accelerometry, global positioning system [GPS]), now allowing for the tracking of a range of objectively measured health behaviors (eg, walking and flu clinic visits). This may increase intervention effectiveness as rewards tied to objectively measured behaviors tend to work better than rewards contingent on self-reported ones [16]. Finally, there are several program design features that can be manipulated to optimize incentive effectiveness (eg, timing, type, magnitude, probability, and schedule), and loyalty points (ie, points given by retailers to promote customer loyalty) have emerged as a promising new incentive “type” (loyalty points vs cash, vouchers or health insurance premium reimbursements or discounts) [26-28]. Not only are Canadians avid loyalty point collectors (90% members of one or more loyalty programs) [29], but the perceived value of their loyalty points may be inflated (in part, because it is not clear how much a single point is actually worth), lowering the reward magnitude needed to stimulate behaviors [16,30-32]. These intervention components may be particularly appealing for governments looking to deploy incentives as efficiently as possible.

The Carrot Rewards app is a new multisectoral mHealth initiative that harnesses the pervasiveness of smartphones and Canadians’ affinity to loyalty programs to reward healthy behaviors. Developed as part of a public-private partnership [33], one of the distinguishing features of the app is its ability to immediately reward users with loyalty points. Specifically, the app rewards Canadians with loyalty points (to go to the movies or grocery store, for gas or travel) for downloading the app, referring friends, and completing an average of 1 to 2 short educational health quizzes each week (“micro-learning”), with the ultimate goal of increasing health knowledge and promoting healthy behaviors. The primary objective of this process evaluation, the first step of a multi-stage evaluation, is to determine the uptake (eg, downloads, completed registrations) of the new app during its 3-month launch period in British Columbia, Canada. The secondary study objectives are to describe the sociodemographic and health behavior characteristics of Carrot Rewards’ users as well as participation levels (eg, proportion of quizzes completed, number of friends referred). A qualitative analysis of app store reviews is also presented.

## Methods

### App Overview

#### Background

Carrot Insights Inc is a private company that developed the Carrot Rewards app in partnership with the Public Health Agency of Canada. British Columbia was the company’s founding provincial partner (the federal-provincial funding arrangement is described elsewhere) [33]. Carrot Insights Inc partnered with 4 major Canadian loyalty programs to offer a variety of popular incentives (ie, points can be redeemed for groceries, air travel, movies, or gas). The company also partnered with 4 Canadian health charities to assist with the development and approval of educational health quiz content (ie, Heart & Stroke Foundation of Canada, Diabetes Canada, YMCA Canada, and the British Columbia Healthy Living Alliance). The marketing assets of 4 loyalty and 1 charity partners were also leveraged such that in the initial weeks of the app launching in British Columbia, partners sent 1.64 million emails to their members (of which 800,167 could be “tracked,” representing the email campaigns of 3 out of the 5 partners).

#### Registration

Carrot Rewards was made available on the iTunes and Google Play app stores on March 3, 2016 in both English and French (Canada’s official languages). Upon downloading the app, users were asked to enter their age, gender, postal code, and loyalty program card number to complete registration (the card of their choice: for either the movies, gas, groceries, or travel loyalty program). To successfully register, users must have entered a valid BC postal code and be 13 years or older (age cut-off of participating loyalty programs). After registration was completed (and a reward worth about US $0.74 earned), a unique promotional code was provided to each user. This code could be shared with “friends;” if a “friend” downloaded the app using the unique code, both parties received bonus points (again worth US $0.74). British Columbians could download the app in one of three ways: organically (ie, finding it in the app store on their own), via partner email invitation, or by using the promotional code “friend referral” mechanism.

#### Intervention

**Figure 1:**
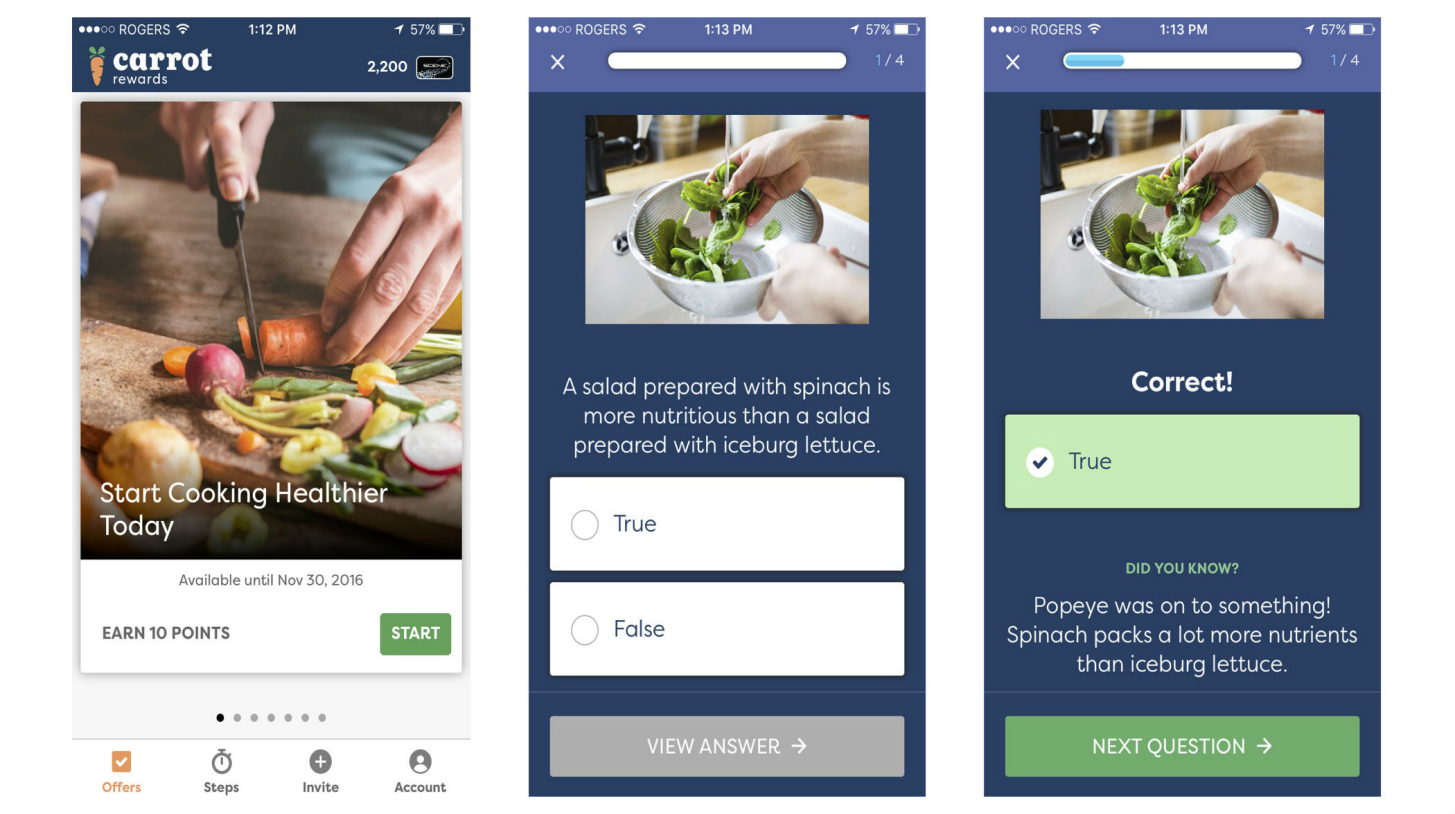
Carrot Rewards app health quiz screenshots (quiz title, question and answer page examples; ‘link out’ screen not shown).

Once the app was downloaded and registration was completed, users were “offered” an average of 1 to 2 educational health quizzes per week, each containing 5 to 7 questions related to healthy eating, physical activity and sedentary behavior, smoking, low-risk drinking, mental health, and immunization—public health priorities identified by the BC Ministry of Health (see colorful and visually appealing screenshots in Figure 1 and quiz “schedule” in Multimedia Appendix 1). In addition, quizzes were developed to inform and familiarize users about self-regulatory health skills or “stepping stone” behaviors (ie, goal setting, tracking, action planning, and barrier identification), skills that have promoted health behaviors in the past [34]. After completing every question in a health quiz and immediately earning incentives (US $0.04 to US $1.48 depending on the length and timing of quiz, ie, earlier quizzes were worth more to stimulate program interest), users could view relevant health information on partner websites. Each health quiz was designed to take approximately 1-3 min to complete. Notably, quizzes were “released” in campaigns (each campaign included about 4 quizzes, so that only users who completed the first quiz in a campaign received subsequent quizzes). In addition to health quizzes, users could earn more points for completing separate health risk assessments (HRAs; Multimedia Appendix 2, which included items from national health surveys (regarding physical activity, eating and smoking habits, alcohol consumption, mental health and overall well-being, as well as frequency of influenza immunization). HRAs were made available in the first 4 weeks of the program (ie, “Carrot Health Survey, 1” and “Carrot Health Survey, 2”). For a full description of the Carrot Rewards intervention, including incentive design features, see Multimedia Appendices 3 and 4. Notable intervention changes during the 3-month evaluation period include gradual reductions in (1) quiz frequency (from about 15 per month to 5 per month, depending on the date a user downloaded the app) as well as (2) reward magnitude (reduction to about 5% of original reward value). Quiz frequency and reward magnitudes were set high initially in an attempt to maximize interest and early participation; both were reduced over time to ensure intervention spending did not outpace the finite budget.

#### Theoretical Rationale

Whereas the Carrot Rewards app is grounded in behavioral economics, broader theoretical considerations regarding how rewards motivate human behaviors may further improve the effectiveness of this approach. Self-determination theory (SDT) is a global theory of motivation that focuses on the extent to which behaviors are controlled by external agents (eg, physicians) or contingencies (eg, rewards) [34]. Whereas behavioral economics describes how rewards can be used to exploit the “present bias” and be a catalyst for change, SDT describes the conditions under which rewards may promote quality, sustained change [34-36]. It was hypothesized that an SDT-informed approach to knowledge building (eg, focus on enjoyment and self-regulatory skills such as goal setting) may help users “internalize” the reasons to engage in healthy behaviors, and in doing so, increase the potential for longer-term change (see Multimedia Appendix 5 for an overview of SDT and its application).

#### Procedure

**Figure 2:**
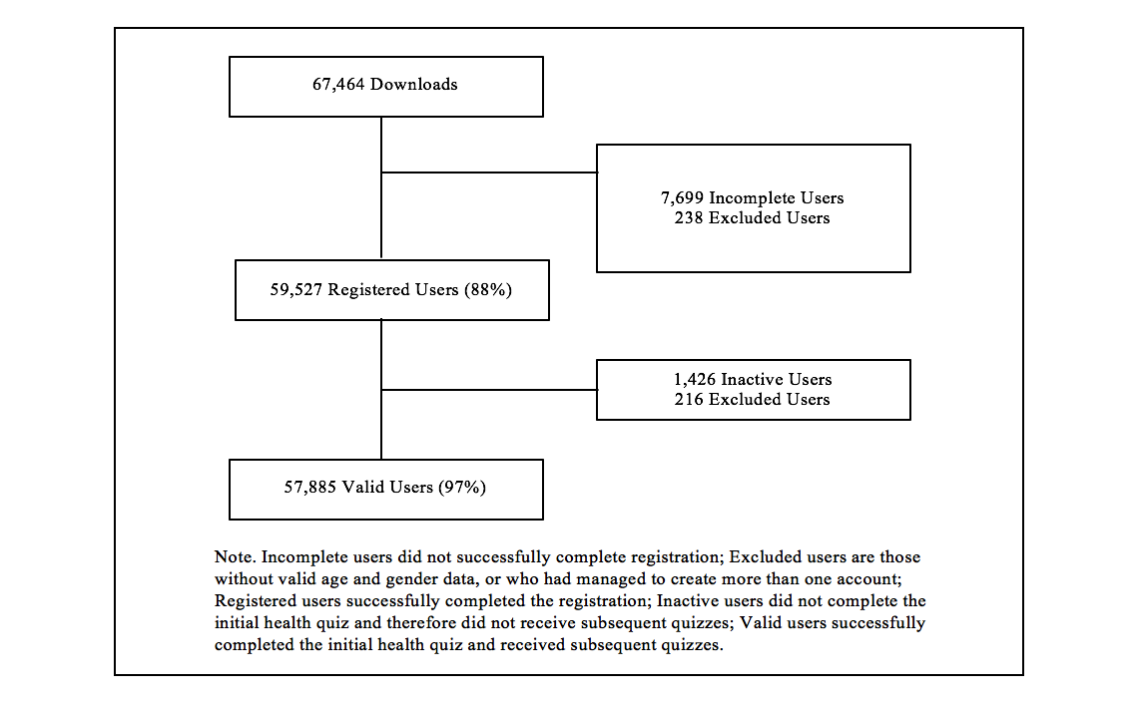
Flowchart of Carrot Rewards app users.

A flowchart is presented in Figure 2 outlining the flow of users through the program: from download to registration to valid user (ie, completed the initial quiz). “Incomplete” users did not successfully complete registration. “Excluded” users are those without valid age and gender data, or who had managed to create more than one account. “Registered” users successfully completed the registration. “Inactive” users did not complete the initial health quiz and therefore did not receive subsequent quizzes. Finally, “valid” users successfully completed the initial health quiz and received subsequent quizzes. App usage data was also collected during the inaugural 3-month period in British Columbia. Research ethics board (REB) approval was not required as the University Health Network REB did not consider this project as research, as described in the Tri-Council Policy Statement V.2, and therefore, did not fall under their purview. The University Health Network REB retrospectively issued an ethics waiver letter (#16-0129) on December 22, 2016. Additionally, as part of the Carrot Rewards’ privacy policy (agreed to by users upon registration), users were informed that data collected in the app for reporting purposes would only be done at the aggregate or deidentified level.

### Data Collection

#### Sociodemographic and Health Behavior Characteristics

In addition to providing information on age and gender, other sociodemographic information was inferred by linking user postal codes with census data (ie, National Household Survey and British Columbia Geographic Service Areas data) at the local health area (LHA) level—there are 89 LHAs in British Columbia. Specifically, median personal income, postsecondary educational attainment, proportion of population identifying as a visible minority, and population density data (metropolitan, urban-rural, rural, and remote) based on LHA were matched to individual users. Users could also complete the HRAs for points. HRA items were modified from valid and reliable questionnaires (eg, Canadian Community Health Survey; Canadian Health Measures Survey) to fit the 15-word app limit (see Multimedia Appendix 2 for HRA items and their source).

#### Participation

App usage data included: individual-level quiz completion data, number of quiz “link outs” clicked, and number of successful friend referrals. Users’ participation levels were classified in the following way: low (<25% of quizzes completed), medium (25- 49% completed), high (50- 74% completed), or very high (>75% completed). Aggregate “app uninstall” data is also reported.

#### Statistical Analyses

Descriptive statistics for sociodemographic (self-reported and inferred from postal code) and health behavior variables are provided. Users with missing age and gender data were excluded from this analysis (n=454). SPSS version 19.0 was used for all statistical analyses.

## Results

### Uptake

Within the first 3 months, 67,464 individuals downloaded the app (62.10% iTunes, Apple Inc Cupertino, CA; 37.90% Google Play, Google Inc Mountain View, CA). In its first week, the Carrot Rewards app was the most downloaded health app in Canada, although only being made available to the population of British Columbia, which makes up about 13% of the national population [37]. During the launch week, 13.79% of the “trackable” emails promoting the app were opened (110,383/800,167) and 1.20% initiated a “click through” to one of the two app stores (19,339/1,610,167; “click throughs” could be tracked for most emails). The majority of downloads occurred organically (46.00%; 31,033/67,464) or via friend referral (31.80%; 21,454/67,464). Among those who downloaded the app, 59,527 (88.23%) individuals successfully completed the registration (ie, registered users). Among registered users, 57,885 (97.24%) were classified as “valid,” having completed at least the initial heath quiz. The Carrot Rewards uninstall rate (data available for Android devices only) was 17.00% (4,518/26,576) during the 3-month study period.

### Sociodemographic and Health Characteristics

Among valid users (n=57,885), the majority were female (62.96%) and aged 18 to 34 years (54.34%; Table 1). More than half of users (52.40%) resided in LHAs where the median personal income was below the provincial average (Can $28,765). Sixty-four percent of users lived in a metropolitan area (LHAs with at least 190,001 residents), compared with 56.17% of the general BC population. The most prevalent chronic disease risk factors were “not” meeting physical activity guidelines (72.70% vs 78% of Canadians in general; Multimedia Appendix 2) and not getting the influenza vaccine in the past year (67.69% vs 69% of Canadians in general; Multimedia Appendix 2). Nine percent of users reported smoking, compared with 14% of all British Columbians (Multimedia Appendix 2).

**Table 1:** Sociodemographics of Carrot Rewards users and British Columbians in general.

Sociodemographics			Carrot Rewards	British Columbia
			n (%)	n (%)
**Gender**			N=57,885	N=4,683,139^a^
	Female		36,446 (62.96)	2,358,862 (50.37)
	Male		20,179 (34.86)	2,324,277 (49.63)
	Prefer not to answer		1,044 (1.80)	Not applicable
	Other		216 (0.37)	Not applicable
**Age (years)**			N=57,885	N=4,683,139^a^
	13-17		1,391 (2.40)	249,064 (6.08)
	18-24		11,954 (20.65)	443,889 (10.84)
	25-34		19,505 (33.69)	634,709 (15.50)
	35-44		11,642 (20.11)	611,064 (14.92)
	45-54		7,625 (13.17)	683,346 (16.69)
	55-64		4,182 (7.22)	653,766 (15.96)
	65-79		1,502 (2.59)	605,065 (14.77)
	80-99		84 (0.15)	212,999 (5.20)
**Income**			N=57,885	N=3,722,755^b^
	Users in LHAs^f^ where the personal median income is below the BC average, n (%)		30,332 (52.40)	1,939,170 (52.09)
**Postsecondary education**			N=57,885	N=2,029,760^c^
	Users in LHAs with fewer residents than average with a postsecondary education		28,788 (49.73)	926,550 (45.64)
**Visible minority**			N=57,885	N=1,180,845^d^
	Users in LHAs with fewer residents than average identifying as a visible minority, n (%)		49,854 (86.13)	951,220 (80.55)
**Population density**			N=57,885	N=4,624,660^e^
	Users living in LHAs categorized by population density, n (%)			
		Remote	296 (0.51)	67,257 (1.45)
		Rural	2272 (3.93)	496,865 (10.74)
		Urban or rural	18,026 (31.14)	1,462,866 (31.63)
		Metropolitan	37,291 (64.42)	2,597,672 (56.17)

^a^Values based on British Columbia’s 2015 population estimates.

^b^Values based on British Columbia’s 2011 National Household Survey. BC personal median income is Can $28,765.

^c^Values based on British Columbia’s 2011 National Household Survey. The proportion of the BC population who reported a postsecondary education is 56%.

^d^Values based on British Columbia’s 2011 National Household Survey. The proportion of the BC population who reported as a visible minority is 38%.

^e^Values based on 2012, 2016 British Columbia Geographic Service Areas data. Remote is classified as a population of 0-10,000 people; rural as 10,001-40,000 people; urban or rural as 40,001-190,000 people, and metro as 190,001+.

^f^LHAs: local health areas.

When asked “What is (are) your biggest health priority(s) in the next 6 months?” in a week 8 quiz, the most commonly cited priorities were: increase physical activity (71.00%; 26,239/36,956), improve eating habits (67.97%; 25,119/36,956), and manage stress levels (41.50%; 15,340/36,956). Finally, when users were asked whether they had learned anything in the first 8 weeks of the program, 93.99% (35,008/37,243) replied “yes.”

### Participation

In the first 3 months, 15 health quizzes and both HRAs were sent to users. A total of 879,616 quizzes or HRAs were sent and 690,111 were completed (78.45%; Multimedia Appendix 1). Regarding quiz completion rate, 60.17% (34,834) of valid users were classified as very high engagers (>75% quiz completion rate), 13.75% (7,954) as high (51-75%), 7.01% (4,057) as medium (26-50%), and 18.45% (10,675) as low (<25%) (N=57,885). Forty-three percent of users (24,870/57,885) completed all 15 quizzes. Regarding attrition, an examination of quiz completion rates for the first quizzes of campaigns 2 (64.53% at week 5; 37,243/57,708) and 3 (62.53% at week 10; 35,855/57,338) suggest participation levels persisted, at least in the short-term. Upon quiz completion, users could learn more about each topic by viewing additional Web-based health resources. Users “clicked out” for more information about 4% of the time (26,574 “clicks;” Multimedia Appendix 6). On average, users sent 3.5 email referrals. The acceptance rate for email referrals was 20.35% (17,540/86,194). Twenty-seven percent (9694/36,445) and 6.08% (2216/36,445) of users successfully referred at least one friend, via email and promotional code, respectively.

### Qualitative Analysis

A conventional content analysis [38] of iTunes and Google Play app store reviews was conducted to examine written reviews. All reviews posted from March 3 to June 6 were compiled and categorized by one researcher as either a positive or negative comment, or both (some reviews had both “positive” and “negative” comments). Both iTunes (n=66; 38.8%) and Google Play (n=104; 61.2%) store reviews were examined. Among “positive” comments (n=119), users highly enjoyed receiving loyalty points for participating in the program (31.9%), users liked learning new health information (11.7%), and users described the app as simple and easy to use (6.7%). The majority of “negative” comments (n=141) were regarding problems loading the app (eg, frozen screens, issues with entering contact information; 15.6%), the reduction in loyalty points over time (11.34%; necessary due to budgetary constraints), and referring friends to the app (9.9%; users required to remember and manually enter friend email addresses). At the 3-month mark (end of the study period), the average app store rating was 2.9 stars for the iTunes store and 3.8 stars for the Google Play store, based on 5-point scale rating systems.

## Discussion

### Principal Findings

Despite much promise, the public health potential of financial health incentives has not yet been realized [20,24]. In particular, incentive programs have been limited in their ability to scale and accommodate entire populations (not just for employees with extended health insurance, for example). Pervasive smartphone use and efficient loyalty points-based incentives allow for broader implementation that is not prohibitively costly. This study represents the first in a planned series with a focus on immediate objectives. Intermediate (eg, health knowledge improvements, short-term improvement in physical activity) and longer term (eg, sustained increase in physical activity, social return of investment) objectives will be evaluated at a later date. The main finding in this study was that an mHealth app that rewards users with loyalty points for downloading (and engaging with) the app was readily downloaded. Uptake was high despite only being available in one province. It is believed that the combination of a comprehensive email campaign, the promise of loyalty points that BC residents already use, and the idea of being rewarded to get or stay healthy, drove interest and uptake. Because there was no control group, the isolated effects of the incentives cannot be established. The early results from this program seem to be enhanced by the use of loyalty points, but not without recognizing the effect of other intervention characteristics (such as the private-public partnership, massive marketing effort, visually appealing design, and so on).

The Carrot Rewards app was the most downloaded health app in Canada during its launch week, despite it being available only in one province. Whereas 46% of downloads occurred “organically,” the 2-way referral bonus (if user successfully refers friend and friend downloads app, both get bonus points) increased the number of users, representing 32% of all downloads. Importantly, 88% of users who downloaded the app successfully registered (eg, entered their loyalty card number), suggesting the onboarding procedure was not onerous. The quiz completion rate (78%) was also higher than expected, with 43% of users completing all 15 of the available “quizzes.” In contrast, a similar mHealth app that used loyalty points to increase downloads but not participation found that 85% of its users were categorized as “very low” or “low” engagers in the first month (ie, fewer than 15 completed “challenges”—though the daily “ask” from the user in this case was heavier with quizzes available every day for 30 days) [27]. Likewise, an eHealth platform promoted using loyalty points (for completing an HRA and enrolling) determined that less than 2% of the approximately 42,000 enrollees were using the tool 6 weeks later [26]. These results are not surprising since attrition is a hallmark of eHealth and mHealth interventions [31]; however, the results presented here suggest that modest incentives in the form of loyalty points may help drive engagement (at least over the course of 3 months). In addition, the number of “clicks” to Carrot Rewards partner websites (n=26,574) highlight the potential role of driving traffic to partner resources.

Whereas the app only required users to report age and gender to minimize friction during registration, supplementary sociodemographic information inferred from postal codes suggests that the current sample is broadly representative of British Columbians in general. Specifically, Carrot Rewards’ captured users in both lower and higher income areas as well as in metropolitan and more rural areas across the province. These finding are consistent with the literature suggesting that lower income adults are especially sensitive to incentive interventions and likely to respond by signing-up for health interventions they may not have otherwise [39-41]. The Carrot Rewards’ user base—in the first 3 months—was predominantly female and between the ages of 18 and 34 years. The incentive-based eHealth and mHealth interventions mentioned earlier report similar demographic profiles, with women especially being more likely to adopt and engage with these interventions (68% and 74% female, respectively) [26,27]. To attract more men and older adults to the platform, several approaches have been recommended, including conducting interviews and focus groups to learn more about intervention components that might appeal to these harder to reach groups (eg, recruitment techniques, loyalty program offerings, and behaviors targeted) [42]. Varying recruitment methods (essentially broadening the “entry points” beyond just email marketing and friend referral) to involve the health care system and specifically leaders within the system (eg, physicians) in the recruitment process (for instance, as a chronic disease self-management tool), may be one way of broadening appeal to under-represented, higher-risk subpopulations.

There are several practical implications of this study. First, though some argue that target groups may disagree with the incentive approach, citing that governments, for example, should not be paying their citizens to engage in healthy behaviors (ie, opportunity cost, paternalism—“nanny state” concerns) [43,44], little evidence of this was uncovered in the quantitative or qualitative aspects of this study. Additionally, the app store ratings at the end of the evaluation period were lower than expected (initial and current ratings are higher—eg, 4+ stars), suggesting that the drop in health quiz frequency and reward magnitude, as well as early technical issues (eg, frozen screens), may have antagonized users. In the future, quiz frequencies and reward magnitudes should be set at levels that can be titrated up (if budgets allow, for example) rather than down (or intermittent reenforcement should be used). It is worth noting that Apple and Google Play store ratings differed (2.9 vs 3.8 out of 5, respectively), possibly because app refinements (eg, bug fixes) occurred at different rates for the iOS and Android versions of the app. Next, the app was successfully launched in large part because of the public-private partnerships that initiated its development. In particular, leveraging the marketing and health content assets of private and government or charity sector partners, respectively, while offering a new way for these partners to communicate with the general population, created a win-win situation for the parties involved. Lessons learned in the multiple-stakeholder development and delivery of the app will be applied as the app prepares to launch in other Canadian provinces or territories and in other sectors (eg, financial literacy). Whereas the preliminary results show that the use of financial incentives encourages engagement with a healthy behavior app supported via a public-private partnership, it does not provide evidence to support the notion that governments should necessarily sponsor such programs. Another practical implication of this work may be in demonstrating that smartphones can be used as vehicles for “immediate” health-related feedback and rewards on a population-scale and that loyalty points in particular may be a useful incentive “form,” given that (1) users are already familiar with the loyalty programs (not a new “currency”), (2) users tend to overvalue these points (when compared with actual dollars) [16,30-32], and (3) users collect points in a variety of ways (not just by using the app), and so are happy for the app to contribute to their growing rewards “pool.” Others looking to maximize the efficiency of their health incentive interventions may be able to apply some of the lessons learned here. The main theoretical contribution of this study may be that incentives need not be prohibitively large or costly to stimulate behaviors on a population-scale; this intervention aspect might actually protect against the often-cited risk of incentive intervention, which is that they undermine intrinsic motives, particularly when targeting health behaviors, rather than just “stepping stone” ones (eg, education) [34-36]. In this case, incentives were deployed not to provide a controlling function (eg, if incentives are very large) but rather an informative one (eg, feedback in the form of modest rewards and health information with every interaction), and may serve to support rather than “crowd out” motivation.

### Limitations

This study is not without limitations. This analysis includes data from British Columbia only, and thus may not be generalizable to other regions that may join in the future. Also, data for this study was extracted before potentially fraudulent user accounts were deactivated. Regarding promotional code use (for referring friends), it was impossible to track number of times the unique codes were shared, as they could be shared on any number of social media sites (or other ways); only number of times promotional codes were “redeemed” were reported. App “uninstall” data was available for Android device users only, which represented only a 38% share of all devices used to download the app. Whereas the sociodemographic data inferred from users’ postal codes is valuable to characterize the user base, strong conclusions cannot be drawn regarding these important sociodemographics. Furthermore, whereas HRA items were adapted from national surveys, their psychometric properties were not tested before implementation; there is evidence of concurrent validity though, with physical activity, flu shot, smoking, and mental health responses aligning with provincial and/or national statistics. The fruit and vegetable items in particular may have been subject to “anchor bias” where users tend to avoid extreme responses (eg, consuming 0 or 5 green veggies yesterday). In the current context, this may have led to overreporting of the number of times fruits and vegetables were consumed yesterday. The overall quiz completion rate (78%) may also have been inflated as users must have completed the first quiz in a “campaign” (a “campaign” is usually 4 quizzes long) in order to receive subsequent quizzes; so, completion rates for quizzes 2-4 in a campaign for example may be inflated since nonadherers do not receive them. Regarding “clicks” to partner websites, whereas 4% of the time users clicked for more information, it is not clear how much time users spent on these sites. Further examination of session times on partner sites is likely required. Whereas the qualitative analysis followed Hsieh and Shannon’s (2005) published framework, it was only conducted by 1 researcher which may limit objectivity of the findings. At most, the result of this qualitative analysis is concept development. Further work is required to increase confidence that conclusions accurately portray the data (eg, employing qualitative data analysis software, peer debriefing). Whereas this study did not examine the impact of quiz frequency and reward magnitude reductions on participation levels over time, future research should explore this issue as well.

### Future Directions

In the future, longitudinal analyses will be conducted to examine the impact of the intervention on changes in self-reported health behaviors as well as health knowledge (baseline vs follow-up). Studies exploring healthy living resource awareness (eg, helpline, Web-resources), self-regulatory skill practices (eg, goal setting, tracking), and objectively measured behaviors (eg, personalized walking goals, getting the flu shot) are planned as well since these are more proximal to the behavioral, health and/or health care system outcomes of interest. A more comprehensive understanding of the contextual factors (eg, demographics) and program features (eg, reward size, probability), as it relates to incentive program effectiveness, would be useful in informing program design in the future. In addition, qualitative work to ascertain intervention components that would appeal to harder to reach subgroups are needed, as are an exploration of new approaches to recruiting users. This study provides further evidence that incentives may be used to stimulate health-related behaviors, though more work is required to elucidate the conditions under which incentives can be used to drive “longer-term” changes. Longer-term cohort studies and other research designs are needed that can attribute behavioral, health, and health economic outcomes to incentive-based mHealth interventions within complex systems of health care.

### Conclusions

The Carrot Rewards app has started to address the problem of scaling up incentive programs while maintaining fidelity to behavioral economics (“present bias;” little delay between behavior and reward for maximum effectiveness). Early results suggest that loyalty points may be used to promote the uptake of an mHealth app in a sample that broadly reflects the sociodemographic and health behavior profiles of British Columbians. A major challenge in mHealth is to develop innovative personalized interventions that can help individuals “maintain” healthy lifestyles and this should be the focus of future work. Their effects on behavioral outcomes (eg, steps per day, flu shot) should be explored, given recent advances in smartphone capabilities. Smartphones in general offer the potential to gather large amounts of data that can be used to better inform interventions. Moving forward, rich datasets should be used to drive the sustained changes needed to produce clinically and economically significant health outcomes.

## Appendix

Multimedia Appendix 1 Carrot Rewards app quiz schedule and completion rates.

Multimedia Appendix 2 Health behaviour characteristics of Carrot Rewards users.

Multimedia Appendix 3 Description of the Carrot Rewards app using the behavioral intervention technology (BIT) model.

Multimedia Appendix 4 Incentive Intervention Framework to describe the Carrot Rewards incentive program.

Multimedia Appendix 5 Application of self-determination theory in the development of the Carrot Rewards app.

Multimedia Appendix 6 Carrot Rewards app end-of-quiz partner website “link out” or “click.”.

## Abbreviations

HRA: health risk assessment
LHA: local health area
REB: research ethics board
SDT: self-determination theory
