# Supplementary material for: Uptake of an Incentive-Based mHealth App: Process Evaluation of the Carrot Rewards App
Source: JMIR Mhealth Uhealth. 2017 May 30;5(5):e70. doi: 10.2196/mhealth.7323 (PMC5470010; doi:10.2196/mhealth.7323)
Supplement: Multimedia Appendix 1 [file mhealth_v5i5e70_app1.pdf]

Quiz Title	Week Sent	Expired[a]	Sent	Completed	Completion Rate
Welcome to Carrot[b]	Onboarding	Yes	59,527	57,885	97%
What Does Eating A Rainbow Taste Like?	Onboarding	Yes	57,485	55,299	96%
No Gym Or Equipment Needed	Onboarding	Yes	57,473	50,747	88%
Stand Up For Your Health	Week 1	Yes	57,456	46,013	80%
Carrot Health Survey, 1	Week 1	Yes	57,051	44,739	78%
Rethink Sugary Drinks	Week 2	Yes	57,450	44,528	78%
The 2 Colours You Shouldn't Eat Without	Week 3	Yes	57,470	42,318	74%
Is Exercise Really Like Medicine?	Week 3	Yes	57,445	41,398	72%
Carrot Health Survey, 2	Week 4	Yes	57,070	37,646	66%
Change Is In The Air[b]	Week 5	Yes	57,708	37,243	65%
Think Small	Week 6	Yes	36,872	32,716	89%
Small Is The New Big	Week 7	Yes	36,872	32,027	87%
Is Sodium Playing Hide And Seek With You?	Week 8	Yes	36,872	31,184	85%
Can You STAND UP For Yourself[b]	Week 10	Yes	57,338	35,855	63%
Do You Know Your Limits?	Week 10	No	26,073	23,381	90%
Sugar Shockers	Week 11	No	26,073	22,624	87%
Physical Literacy: The Big Picture	Week 13	No	26,073	21,955	84%
Second-Hand Smoke: Is It Really A Big Deal[b]	Week 14	No	57,308	32,553	57%
**Total**			**879,616**	**690,111**	**78%**

Note. [a]All quizzes have a 30-day expiry date; 'No' represents data being extracted prior to the 30-day expiry date. [b]Quizzes represent the start of a new 'campaign'. Users must complete the first quiz in order to receive subsequent quizzes.
