# Supplementary material for: Uptake of an Incentive-Based mHealth App: Process Evaluation of the Carrot Rewards App
Source: JMIR Mhealth Uhealth. 2017 May 30;5(5):e70. doi: 10.2196/mhealth.7323 (PMC5470010; doi:10.2196/mhealth.7323)
Supplement: Multimedia Appendix 2 [file mhealth_v5i5e70_app2.pdf]

**Health Risk Assessment, Part 1**, *N* = 44,739	**Adapted from Source Survey**	**n**	**%**
In the past 7 days, how much time did you spend doing activities that made you sweat at least a little and breathe harder? Only count the activities that lasted 10 minutes or longer[a]	IPAQ-SF		
Less than 2.5 hours		31,765	73%
Yesterday, how many times did you eat fruits and veggies[b]	CCHS 2015		
Less than 5 times		16,975	38%
Yesterday, how many times did you have a sugary drink like pop, sweetened tea or coffee (hot/cold), bubble tea, vitamin water, or a fruit/sport/energy drink?[c]			
1 or more times		28,082	63%
Thinking about the past 7 days: How often did you eat dinner that was prepared at home?[d]			
Less than 6 times		25,771	58%
When did you have your last flu shot? Don't include the "H1N1" flu shot?[e]	CCHS 2015		
I've never had a flu shot		12,811	29%
More than a year ago[f]		17,475	39%
**Health Risk Assessment, Part 2**, *N* = 37,646		**n**	**%**
Thinking back over the past week, how many "drinks" did you have in total?[g]	CCHS 2015		
Males not meeting low risk drinking guidelines		323	3%
Females not meeting low risk drinking guidelines		658	3%
If you smoke cigarettes, how many do you smoke each day?[h]	CCHS 2015		
1 or more		3,443	9%
Mental wellbeing is a pretty big deal. In general how would you say your mental health is?[i]	CCHS 2015		
Poor or fair		6,198	17%
How do you feel about your life as a whole right now?[j]	CCHS 2015		
Very dissatisfied or dissatisfied		5,751	15%
How would you describe your sense of belonging to your local community?[k]	CHMS 2013		
Very weak or somewhat weak		15,876	44%

Note. IPAQ-SF = International Physical Activity Questionnaire, Short Form; CCHS = Canadian Community Health Survey; CHMS = Canadian Health Measures Survey.

[a]Response options were on a 3-point Likert scale ranging from 1 (less than 60 min [not very active]) to 3 (more than 2.5 hrs [very active)]). Percentages indicate a combined sum of the response options ‘less than 60 min (not very active)’ and ‘between 60 min and 2.5 hrs (fairly active)’ for users 18 years and older. Total sample of users 18 years and older, n = 43,692.
[b]Asked about fruits, dark green veggies, orange veggies, other veggies; Rsponse options were on a 6-point Likert scale ranging from 1 (0 times) to 6 (5 or more times). Percentages indicate a combined sum of the response options ‘0 times’, ‘1 time’, ‘2 times’, ‘3 times’, and ‘4 times’.
[c]Response options were on a 6-point Likert scale ranging from 1 (0 times) to 6 (5 or more times). Percentages indicate a combined sum of the response options ‘1 time’, ‘2 times’, ‘3 times’, ‘4 times’ and ‘5 or more times’.
[d]Response options were on a 8-point Likert scale ranging from 1 (0 times) to 8 (Everyday). Percentages indicate a combined sum of the response options ‘0 times’ ‘1 time’, ‘2 times’, ‘3 times’, ‘4 times’ and ‘5 times’.
[e]Response options were on a 4-point Likert scale ranging from 1 (I've never had a flu shot) to 4 (less than a year ago).
[f]Percentages indicate a combined sum of the response options ‘1 to 2 years ago’ and ‘2 years ago or more’.
[g]Response options were on a 8-point Likert scale ranging from 1 (none, zero, zilch) to 8 (5 or more drinks per day). Percentages indicate a combined sum of the response options ‘4 drinks per day’ and ‘5 or more drinks per day’ for male users, n = 12,605. Percentages indicate a combined sum of the response options ‘3 drinks per day’, ‘4 drinks per day’ and ‘5 or more drinks per day’ for female users, n = 24,266.
[h]Response options were ranged from 0 to 50 cigarettes.
[i]Response options were on a 5-point Likert scale ranging from 1 (poor) to 5 (excellent).
[j]Response options were on a 5-point Likert scale ranging from 1 (very dissatisfied) to 5 (very satisfied).
[k]Response options were on a 5-point Likert scale ranging from 1 (very weak) to 5 (very strong).
