# Supplementary material for: Uptake of an Incentive-Based mHealth App: Process Evaluation of the Carrot Rewards App
Source: JMIR Mhealth Uhealth. 2017 May 30;5(5):e70. doi: 10.2196/mhealth.7323 (PMC5470010; doi:10.2196/mhealth.7323)
Supplement: Multimedia Appendix 3 [file mhealth_v5i5e70_app3.pdf]

The BIT model (next page) was chosen as it provides a framework to inform the development of behaviour change interventions delivered using technology. The BIT model below describes the specific aims and theoretical constructs used in the *Carrot Rewards* app to target behaviour change. The BIT model consists of two levels: 1) a theoretical level, describing the “why” or aims of the intervention, and the “how” or behaviour change techniques used to achieve the intervention’s objectives; and 2) the instantiation level describes the technological components of the intervention, including “what” is being delivered, as well as “how” and “when” the content is being delivered. Relevant behaviour change techniques were selected from Michie et al.’s taxonomy to target the relevant theoretical constructs (i.e. knowledge, competence, self-regulation). The table below also describes the technological features of the app.

	BIT component	Example	Example from *Carrot Rewards* app
Theoretical			
Why	Aims		
		Clinical aims	Improve knowledge of healthy lifestyle practices<br>Improve healthy lifestyle behaviours
		Usage aims	Download/Register<br>Participation (complete quizzes, click quiz ‘link outs’, refer friends)
How (conceptual)	Theoretical constructs		Behaviour Change Techniques
		Behavioural economics	10.2 Material reward for a behaviour
		Knowledge of health risks and benefits/stage of change	4.1 Instruction on how to perform a behaviour<br>5.1 Information about health consequences<br>5.6 Information about emotional consequences<br>4.2 Information about antecedents
		Decisional balance/outcome expectation	9.2 Pros and cons<br>9.1 Credible source
		Self-efficacy/control	8.7 Graded tasks<br>15.1 Verbal persuasion about capability
		Self-regulation/goals/ intention/planning	2.3 Self-monitoring of behaviour<br>8.2 Behaviour substitution<br>1.4 Action planning<br>1.2 Problem solving<br>1.8 Behavioural contract<br>1.9 Commitment<br>1.1 Goal setting (behaviour)<br>7.1 Prompts/cues
		Social support/subjective norm	3.1 Social support (unspecified)
Instantiation			
What	Elements		
		Information delivery	Text, image and videos in quizzes
		Notifications	Within app and push notifications that new quizzes are available
		Logs	Self-reported health behaviours captured
		Passive data collection	Phone sensor (built-in accelerometer/geo-locating – to be launched after initial 3-month period)
How (technical)	Characteristics		
		Medium	Text, images and videos
		Complexity	Simple drop down menus
		Aesthetics	Visually appealing with images and limited text per screen
When	Workflow		
		Frequency	Quizzes are made available every 3-4 days
		Conditions	Task-based, new content released after previous quizzes completed
