# Supplementary material for: Uptake of an Incentive-Based mHealth App: Process Evaluation of the Carrot Rewards App
Source: JMIR Mhealth Uhealth. 2017 May 30;5(5):e70. doi: 10.2196/mhealth.7323 (PMC5470010; doi:10.2196/mhealth.7323)
Supplement: Multimedia Appendix 4 [file mhealth_v5i5e70_app4.pdf]

**Domain**	**Dimension**
Direction	Positive reward after completing each quiz
Form	Loyalty program reward points (groceries, movies, gas, air travel)
Magnitude	Range: $0.05 to $1 depending on the length and timing of quiz (earlier quizzes were worth more to stimulate program interest)
Certainty	Certain. Points are always awarded upon quiz completion, as are successful friend referral
Target	Process. Points are awarded for completing quizzes that will enhance knowledge of healthy living practices
Frequency	All instances incentivized (1 to 3 quizzes per week)
Immediacy	Immediately upon quiz completion
Schedule	Fixed. Quizzes are worth a pre-specified number of points (magnitude varies)
Recipient	Individual. Points are awarded to the individual participant
