# Supplementary material for: Uptake of an Incentive-Based mHealth App: Process Evaluation of the Carrot Rewards App
Source: JMIR Mhealth Uhealth. 2017 May 30;5(5):e70. doi: 10.2196/mhealth.7323 (PMC5470010; doi:10.2196/mhealth.7323)
Supplement: Multimedia Appendix 5 [file mhealth_v5i5e70_app5.pdf]

According to SDT, behaviours are motivated for different extrinsic reasons that represent varying degrees of 'internalization'.[34] SDT describes a motivation continuum ranging from amotivation (no motivation) to intrinsic regulation with four forms of extrinsic motivation in between - the key difference being the degree to which they vary in terms of self-determination (e.g., "I exercise because it is an important part of who I am" vs. "I exercise because I want to lose weight").[34] SDT also defines intrinsic motivation as the desire to do something for its own sake and in the absence of external controls.[34] Since more self-determined motives are associated with better behavioural outcomes (e.g., daily adherence),[36] this intervention was designed to foster the three basic psychological needs that shape movement along the continuum – (1) competence (sense of mastery), (2) autonomy (sense of ownership), and (3) social relatedness (feeling connected to others). For example, according to SDT, increasing knowledge of healthy living practices could facilitate movement across the motivation continuum.[36] Not just increasing knowledge of health benefits or consequences *per se* but also of the enjoyment inherent in many health behaviours (e.g., dancing, 'eating a rainbow of colours') as well as of the self-regulatory skills known to underpin quality (not controlled) behaviour change (e.g., goal setting, tracking, problem solving).[34] It was hypothesized that this approach to knowledge building might increase feelings of competence ("Hey, I can do that!") and autonomy ("My top health priority this year will be…"). To further initiate movement along the continuum, a two-way referral system was employed to encourage users to invite their friends to participate, potentially increasing feelings of social relatedness as well. Taken together, the intervention focus during this initial 3-month launch period was on building health knowledge and in doing so try to satisfy the three needs using a simple, fun and engaging quiz format where rewards provided an informational (i.e. feedback given with every interaction) rather than a controlling role.[34,36]
