# Supplementary material for: Uptake of an Incentive-Based mHealth App: Process Evaluation of the Carrot Rewards App
Source: JMIR Mhealth Uhealth. 2017 May 30;5(5):e70. doi: 10.2196/mhealth.7323 (PMC5470010; doi:10.2196/mhealth.7323)
Supplement: Multimedia Appendix 6 [file mhealth_v5i5e70_app6.pdf]

**Quiz Title**	**Week Sent**	**Clicks**	**Click-through Rate**
Welcome to Carrot	Onboarding	-	-
What Does Eating A Rainbow Taste Like?	Onboarding	3,401	6%
No Gym Or Equipment Needed	Onboarding	2,828	6%
Stand Up For Your Health	Week 1	2,185	5%
Carrot Health Survey	Week 1	-	-
Rethink Sugary Drinks	Week 2	2150	5%
The 2 Colours You Shouldn't Eat Without	Week 3	2,954	7%
Is Exercise Really Like Medicine?	Week 3	1,500	4%
Carrot Health Survey, 2	Week 4	-	-
Change Is In The Air	Week 5	2,278	6%
Think Small	Week 6	1,568	5%
Small Is The New Big	Week 7	1,724	5%
Is Sodium Playing Hide And Seek With You?	Week 8	1,294	4%
Can You STAND UP For Yourself	Week 10	1,509	4%
Do You Know Your Limits?	Week 10	881	4%
Sugar Shockers	Week 11	690	3%
Physical Literacy: The Big Picture	Week 13	849	4%
Second-Hand Smoke: Is It Really A Big Deal	Week 14	763	2%
**Total**		**26,574**	**4%**

Note. '-' no link outs were provided for these quizzes.
